# Control-Relevant Adaptive Personalized Modeling From Limited Clinical Data for Precise Warfarin Management

**DOI:** 10.1109/OJEMB.2023.3240072

**Published:** 2023-01-26

**Authors:** Affan Affan, Jacek M. Zurada, Tamer Inanc

**Affiliations:** Electrical and Computer Engineering DepartmentUniversity of Louisville 5170 Louisville KY 40292 USA; Electrical and Computer Engineering DepartmentUniversity of Louisville 5170 Louisville KY 40292 USA; Information Technology InstituteAcademy of Social Sciences 90-193 Lodz Poland

**Keywords:** Adaptive modelling, drug dosing, model (In)validation, robust system identification, warfarin management

## Abstract

Warfarin is a challenging drug to administer due to the narrow therapeutic index of the International Normalized Ratio (INR), the inter- and intra-variability of patients, limited clinical data, genetics, and the effects of other medications. *Goal:* To predict the optimal warfarin dosage in the presence of the aforementioned challenges, we present an adaptive individualized modeling framework based on model (In)validation and semi-blind robust system identification. The model (In)validation technique adapts the identified individualized patient model according to the change in the patient's status to ensure the model's suitability for prediction and controller design. *Results:* To implement the proposed adaptive modeling framework, the clinical data of warfarin-INR of forty-four patients has been collected at the Robley Rex Veterans Administration Medical Center, Louisville. The proposed algorithm is compared with recursive ARX and ARMAX model identification methods. The results of identified models using one-step-ahead prediction and minimum mean squared analysis (MMSE) show that the proposed framework effectively predicts the warfarin dosage to keep the INR values within the desired range and adapt the individualized patient model to exhibit the true status of the patient throughout treatment. *Conclusion:* This paper proposes an adaptive personalized patient modeling framework from limited patientspecific clinical data. It is shown by rigorous simulations that the proposed framework can accurately predict a patient's doseresponse characteristics and it can alert the clinician whenever identified models are no longer suitable for prediction and adapt the model to the current status of the patient to reduce the prediction error.

## I. Introduction

Warfarin is an oral anticoagulant used to decrease blood clotting and avoid thromboembolic events in the human body. The main reason for the thrombotic events is the blood clots that break loose and are stuck in narrow vessels. The effect of warfarin is monitored by measuring the International Normalized Ratio (INR). INR is the ratio between the patient's prothrombin time (PT) and the mean normal PT [1]. The desired range of INR values is between 2.0 to 3.0 [2]. In addition to the narrow therapeutic range of INR, the INR is highly affected by genetics, change in diet, and the use of other medications throughout treatment. These fluctuations can lead to under-anticoagulation, increasing the risk of clotting, and over-anticoagulation, increasing the risk of release of blood from broken blood vessels [3].

The dosage of warfarin is affected by dietary interactions, drug interactions, demographic effects, and genetic effects [4]. Vitamin K is a natural antidote to warfarin and most dark green vegetables such as broccoli consist of a high level of vitamin K [5]. On average, a human takes around 60–200 $\mu g/day$ of vitamin K, while the intake of 100 $\mu g/day$ of vitamin K for 4 consecutive days can lower the INR value by 0.2 [6]. One of the major factors which influence the INR value in the human body are interactions of other drugs with warfarin [7]. Drugs such as amiodarone used to treat heart rhythm problems interact with warfarin to increase the anticoagulant effect [8]. On the other hand, drugs like aspirin increase the risk of bleeding by eliminating warfarin [9]. Other factors affecting INR values are genes, age, height, smoking, etc [10], [11]. The aforementioned factors lead to the ineffective warfarin dosage, which is the common cause of most hospital visits in the USA and U.K. [12], [13].

The narrow therapeutic range of INR and risk of bleeding due to unintentional overdosing of warfarin urge the need for a robust, adaptive, and patient-specific warfarin management system that represents true patient status in the event of a change in medication or diet [4]. Another challenge for the applications of precise drug delivery is the slow sampling rate as often measurement sampling time is of days and weeks. It can slow the process of data collection and results in small data sets. Therefore, a modeling algorithm is needed that can identify the model at an early stage and recursively improve the model capabilities as the new measurement data is received. Also, due to swift changes in factors related to warfarin, the prediction models must show the true status of the patients using a limited number of available clinical data. Therefore, the model must learn the change in the dose-response characteristics of each patient and adapt to the wavering status within an equitable time frame.

Many researchers have proposed dose-response models to predict the dosage of warfarin using different model identification techniques. Numerous prediction models have been proposed in the literature that use the Bayesian approach to predict the warfarin dosage [14], [15], [16], [17]. The Bayesian approach is prominent in model identification due to its ability to take full account of uncertainties related to the model and parameter values. However, Bayesian methods require the selection of probability distributions of disturbances and noise, which can be time-consuming and may lead to erroneous dose-response models if prior distributions are imprecise [18], [19], [20].

Several artificial intelligence (AI)-based predictive models have been proposed using different methods [21], [22], [23]. The AI-based models can efficiently predict warfarin dosage to reduce the risk of overdosing or under-dosing. The main challenge in AI-based models is the requirement of a large data set, which is a drawback as patient-specific clinical dose-response data for warfarin is usually not available in high volume. Moreover, the AI-based models are typically trained using population-based data sets. This is very pivotal because, with the use of population-based data sets, the inter-and intra-variability among the patients can not be effectively addressed.

In recent years, control systems theory has played a vital role in designing methods to predict optimal drug dose and control the dose-response of several diseases such as diabetes, venous thrombosis, and chronic kidney disease [24], [25], [26], [27], [28], [29], [38], [39], [40], [41]. Control systems theory-based model identification techniques provide the prediction models in the form of the transfer function, state-space, difference, and differential equations. The advantage of control-oriented model identification-based techniques is the ability to provide robustness and guaranteed stability using limited patient-specific dose-response data. However, some classical identification techniques require fixed model structure and priori noise and uncertainty distributions related to the model such as auto-regression and moving average-based model identification techniques. In contrast to classical identification techniques, robust model identification does not require a large measurement data set or information on measurement noise, or information on the structure of the model. The robust identification technique needs a maximum gain value of the model, $K$, the stability margin of the model response, $r$, and a bound on the noise, $\epsilon$ [30], [31], [32], [33], [34], [35].

In this research work, semi-blind robust model identification is used to identify the individualized patient model using a limited number of patient-specific clinical data points, and model (In)validation is implemented for the model adaptation to guarantee the suitability of the identified models for prediction and controller design [36], [37], [39]. For model (In)validation, unused data points of the patient corresponding to the identified model are used and mathematical evidence is provided about the model's suitability to predict warfarin dosage. One-step-ahead prediction is used and minimum mean squared error (MMSE) is calculated between predicted INR values and clinically obtained INR values. To show the significance of model (In)validation, the prediction results of the patient models obtained without model (In)validation are also discussed. The models with model (In)validation are compared with models obtained without model (In)validation by MMSE values.

The rest of this paper is structured as follows: In part II, semi-blind robust system identification is introduced. Section III covers model (In)validation, whereas Section IV gives a pseudo algorithm for semi-blind robust identification using model (In)validation. Section V provides the patient model results produced by one-step-ahead prediction, as well as the error analysis followed by a conclusion.

## II. Semi-Blind Robust Identification

**Fig. 1.**
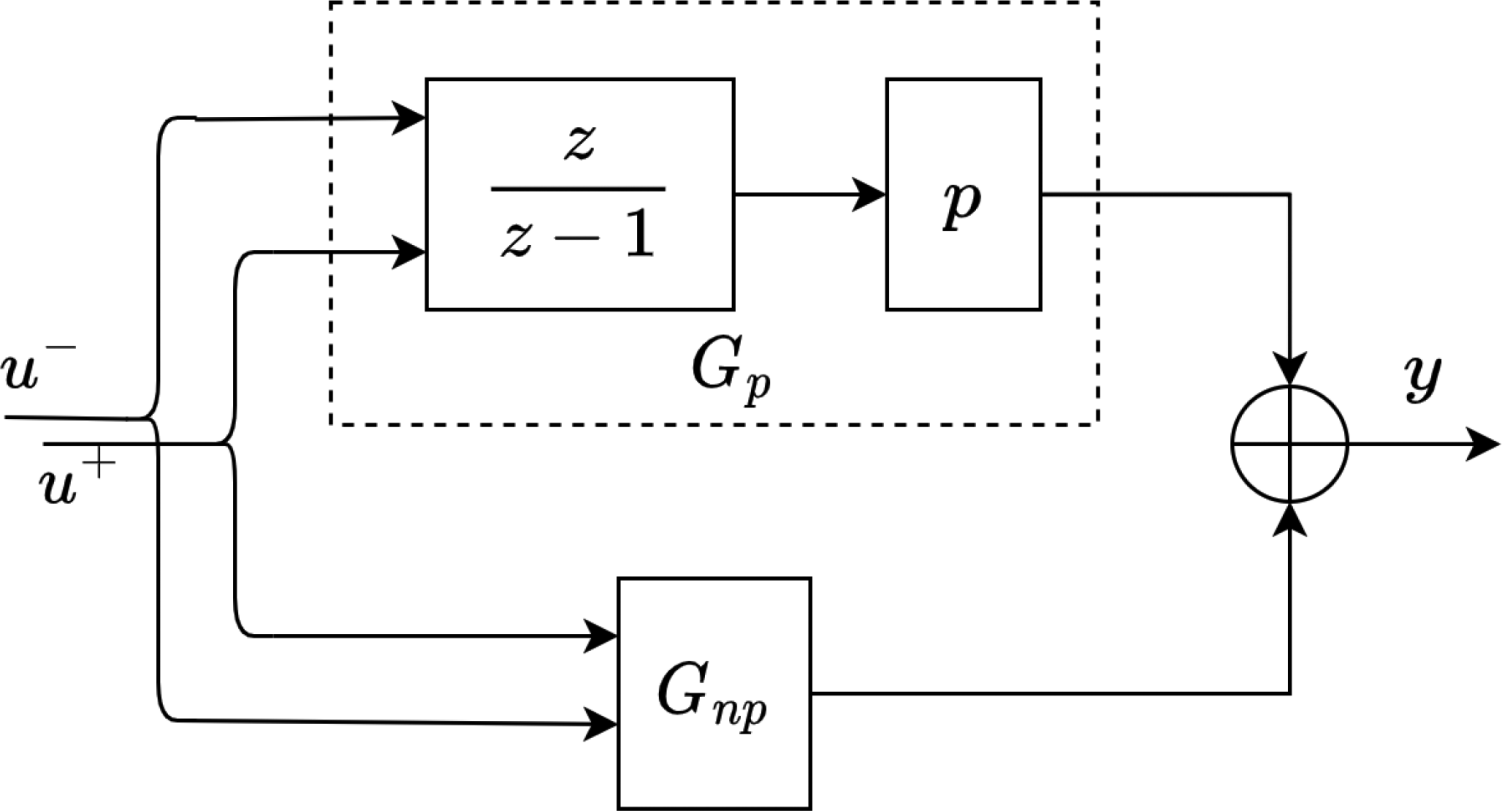
Semi-blind robust system identification framework.

The patient model is the set of mathematical equations that describes a patient's response to a certain amount of drug dosage. Model identification aims to obtain a low-order model of the patient by using a finite, noisy, and limited number of clinical dose-response data. A patient's model consists of parametric and non-parametric portions as shown in Fig. 1. The parametric portion describes the influence of the intermediate variables known as states and the non-parametric portion describes the input-to-output relation. The patient's general model can be written in the following simplified form:

\begin{equation*}
G=G_{p}+G_{np}, \tag{1}
\end{equation*}

where $G_{np}$ is the non-parametric portion and $G_{p}$ is the parametric portion. Typically, model identification is performed with steady-state data and zero initial conditions. However, in the area of drug dosing, the patients have a history of medication for different diseases and due to the early stages of treatment, steady-state data is not always available. Therefore, it is not equitable to consider zero initial conditions for the model identification of patients treated with warfarin. In light of these challenges, semi-blind robust identification is more suitable since it utilizes the effect of non-zero initial conditions in the model identification framework to reduce the identification error. The model identification problem is described below [30].

*Problem 1:* Obtain the patient model $G$ which gives output $y_{i}$ as INR level in response to input $u_{i}$ of warfarin dosage.

\begin{align*}
y_{i}=&Gu_{i}+\eta _{i}, \tag{2}
\\
\left|\eta _{i}\right|\leq & \epsilon _{i}, \tag{3}
\end{align*}

where $\eta _{i}$ is the model noise and $\epsilon _{i}$ is the maximum noise bound.

Now we formally introduce the problem statement for semi-blind robust identification as follows [36], [37]:

*Problem 2:*Determine $G(z)=G_{p}(z)+G_{np}(z)$, which is consistent with priori and posteriori information, such that $\tau$ is a non-empty set, given input sequence $u$, output sequence $y$, noise bound $\epsilon$, maximum stability gain $K$, and features of past input $u^-$. Given the state space model, $G=\begin{bmatrix}A_{g} & B_{g} \\
 C_{g} & D_{g} \end{bmatrix}$, $\tau$ is defined as:

\begin{equation*}
\tau (y)\doteq y_{i}=\sum _{i=0}^{N_{t}}g_{i} u^{N_{t}-i}+C_{g} A_{g}^{N_{t}-1}\left(\Gamma _{g}^{N_{t}}u^-\right)_{i=0} \tag{4}
\end{equation*}

where $g_{0}=D_{g}$ and $g_{i}=C_{g}(A_{g})^{i-1}B_{g}$. $N_{t}$ is the number of data points used for model identification and $\mathrm{\Gamma }_{\mathrm{g}}^{\mathrm{N_{t}}}$ is the Hankel matrix associated with coefficients $g$. Because of the Bi-Affine matrix, Equation (4) is non-convex which can be transformed into a convex problem as explained in [36]. The conversion described in [36] retains the specified model's observability and controllability. The following is a definition of the modified convex problem:

*Problem 3:*Determine that $G(z)=G_{p}(z)+G_{np}(z)$ such that $\tau$ is a non-empty set that is consistent with a priori and a posteriori information [36]:

\begin{equation*}
\tau (y)=\left\lbrace G(z)\in S:y_{i}-\left(T_{g}^{N_{t}} u^+\right)_{i}+\left(\mathrm{\Gamma }_{\mathrm{g}}^{\mathrm{N_{t}}}u^-\right)_{i}\right\rbrace \tag{5}
\end{equation*}

where $|(\mathrm{\Gamma }_{\mathrm{g}}^{\mathrm{N_{t}}}u^-)_{i}|\leq \ \gamma; i=0,1,\ldots,{N_{t}}-1$ and $T_{g}^{N_{t}}$ is the Toeplitz matrix. The initial portion of the $\tau$ set relates to the patient reaction to input $u$, while the latter part gives information about the patient response to previous inputs $u^-$. This problem can be solved by following Linear Matrix Inequalities (LMIs) as discussed in [36]:

\begin{align*}
&M(g)=\begin{bmatrix}KR^{-2} & \left(T^{N_{t}}_{g}\right)^{T}\\
 \left(T^{N_{t}}_{g}\right) & KR^{2} \end{bmatrix}\geq 0,\\
&\left| y-\left(T_{u}^{N_{t}} pP+T_{u}^{N_{t}} g\right)-\Gamma _{g}^{N_{t}}u^{-}\right|\in N,\\
&-\gamma K_{u} \leqslant \Gamma _{g}^{N_{t}}u^{-}\leqslant \gamma K_{u},
\end{align*}

where $\gamma,K_{u},p,\ P$ represent model gain, bound on the norm of sequence $u^-$, affine parameters, and the parametric portion of the model, respectively. As the INR level of the patient is also subject to change by the past inputs $u^-$; i.e initial conditions, the effect of past inputs should be incorporated by an integrator into the parametric portion.

To this point, semi-blind robust model identification is presented to acquire individualized patient models using the minimum number of time-domain clinically acquired patient dose-response data. However, as time elapses the patient's status might change because of maturing, change in food plan, and use of another drug for another illness. Therefore, it is possible that the patient model obtained at an early stage of treatment may not represent the patient's current status anymore and is no longer suitable for dosage prediction. To adjust the model to the patient's present status, the following segment examines the model (In)validation procedure which gives numerical proof of the model's suitability for prediction and controller design by testing the identified model on new unused data of the same particular patient.

## III. Model (In)validation

**Fig. 2.**
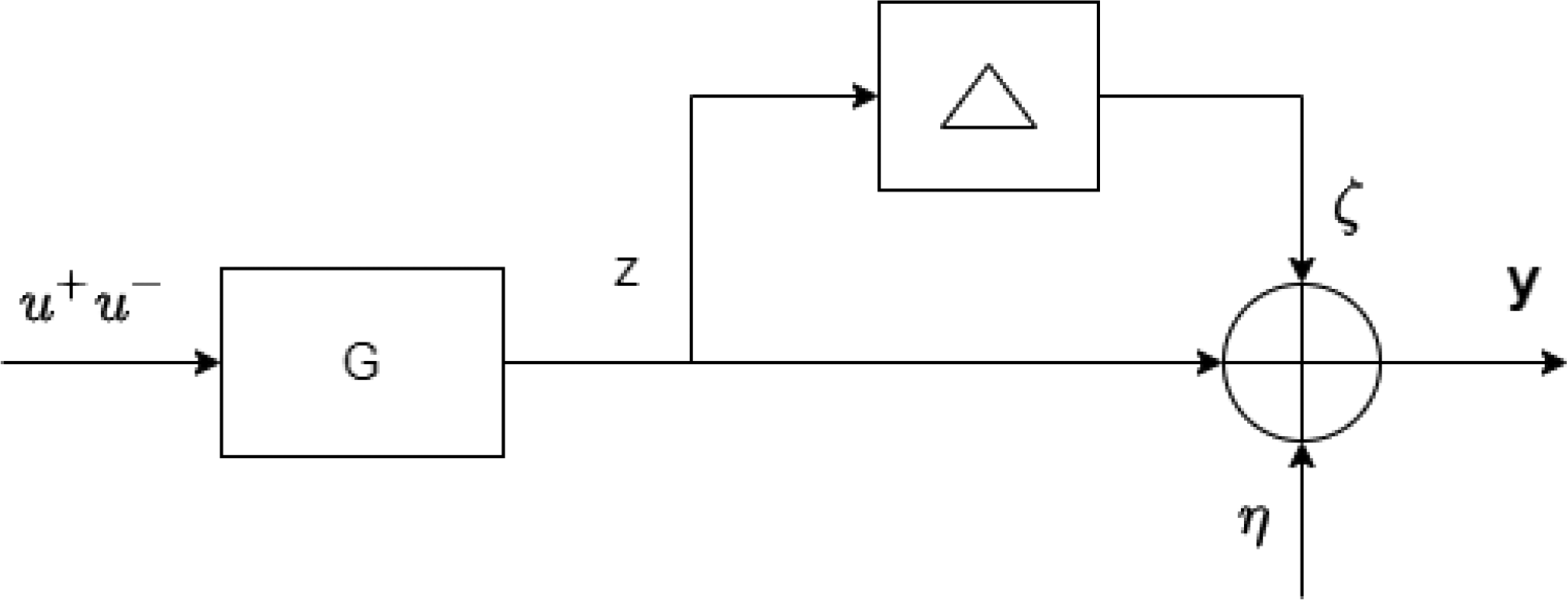
The non-convex framework for model (In)validation.

The semi-blind robust model identification framework utilizes a small amount of patient-specific clinical noisy data, to obtain an individualized patient model. The theory makes the researcher capable to design individualized patient models which are robust and stable in theory but there is no assurance that the models will be stable and robust in practice. This problem arises as a result of the ambiguous model uncertainty and changes in the patient's status throughout therapy. Therefore, the personalized patient models should be validated by using additional patient-specific data that is not used in the identification process. Model (In)validation technique provides confirmation of the model's usefulness for prediction and controller design under these uncertainties by evaluating the model on a new data set of the same patient. The problem is expressed as follows, assuming multiplicative and additive noise as illustrated in Fig. 2.

**Fig. 3.**
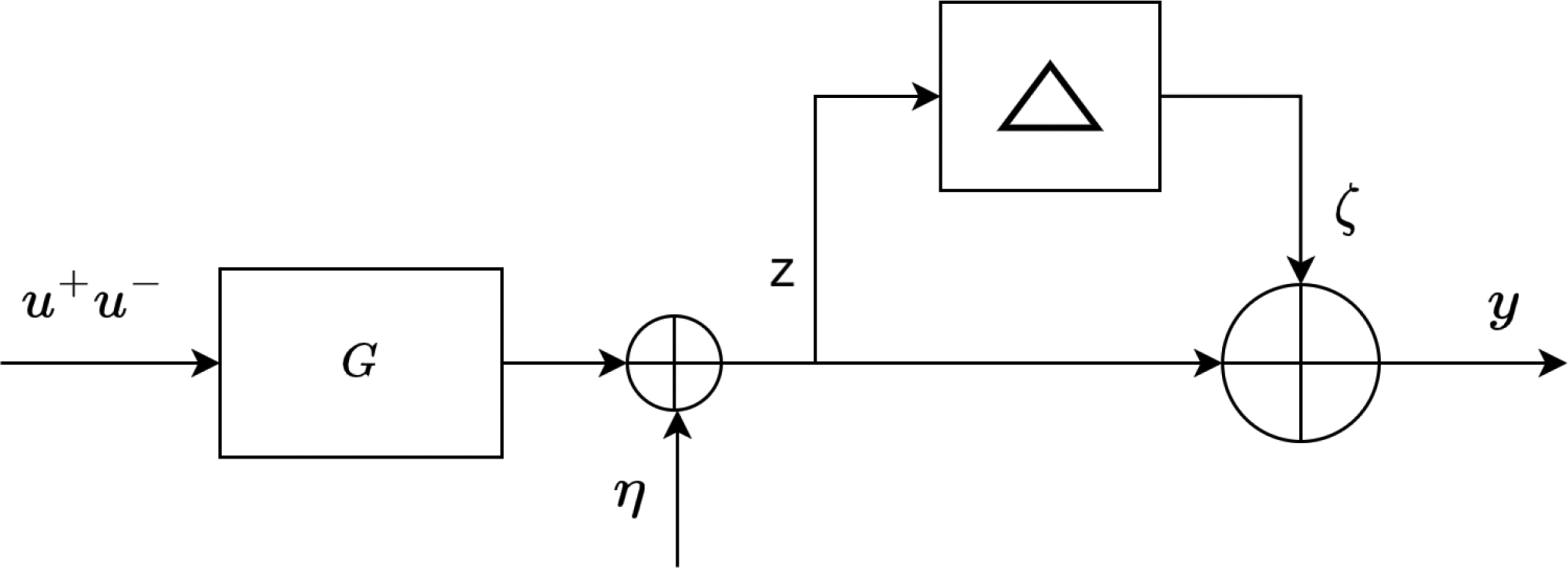
The convex relaxed framework for model (In)validation.

*Problem 4:*Given $M_{t}$ clinical data points, the model $G(z)\in S$, descriptions of admissible noise $\eta$, uncertainty $\Delta$ and initial conditions $x_{0}$, determine if there exists at least one combination of $(\eta, \Delta,x_{0})$ for $\zeta = \Delta z$ that can reproduce the available clinical patient data by the following equation [36]:

\begin{equation*}
y=\left(I+\Delta \right)\left(T_{g}u^++T_{g}^{ic}x_{0}\right)+\eta, \tag{6}
\end{equation*}

where $u^+$ represents the input after $n=0$, $T_{g}$ maps the input to the output, $T_{g}^{ic}$ translates the starting conditions to the output. The term $T_{g}^{ic}x_{0}$ refers to the aforementioned problem, where $x_{0}$ represents the unknown initial condition. This term can be replaced with some term representing the effect of these initial conditions such as $u^-\in \mathcal {U}_-$, therefore, (6) can be modified as follows:

\begin{equation*}
y=\left(I+\Delta \right)\left(T_{g} u^+ +\mathrm{\Gamma }_{\mathrm{g}} u^-\right)+\eta, \tag{7}
\end{equation*}

where $u^-$ and $\mathrm{\Gamma }_{\mathrm{g}}$ denote the past admissible inputs and Hankel matrix, respectively. To avoid problems with the solution of the non-convex problem, the following convex relaxation is explored, as stated in [36]. Fig. 3 shows the alternative setup for the model (In) validation, where measurement noise is also affected by $\Delta, \eta \doteq (1+\Delta)\bar{\eta }$. Equation (7) can be modified as follows:

\begin{equation*}
y=\left(I+\Delta \right)\left(T_{g}u+\mathrm{\Gamma }_{\mathrm{g}} u^-+\ \bar{\eta }\right) \tag{8}
\end{equation*}

Equation (8) is satisfied if a triple $(u^-,\bar{\eta },\Delta)$ exists and $\Vert \Delta \Vert _\infty < 1$. For further details of the convex relaxation of the model (in)validation process, please see [36].

## IV. Adaptive Model Identification Algorithm

Semi-blind robust model identification technique and model (In)validation framework have been discussed in Section II and III, respectively. This section combines these techniques to develop an adaptive model identification method, which improves the prediction accuracy and ensures the identified models are suitable for controller design as well.

**Fig. 4.**
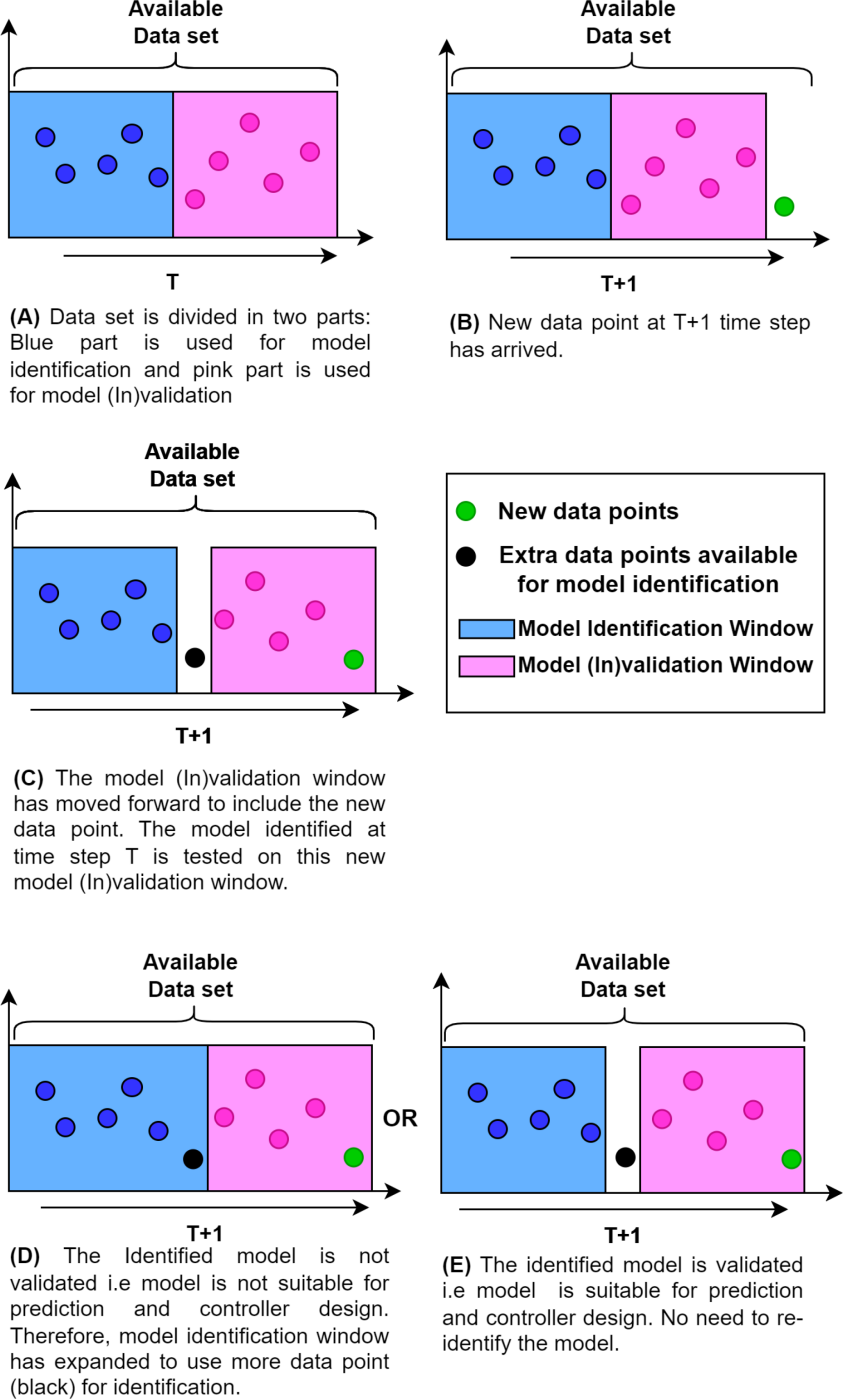
Recursive adaptive model identification.

The general framework of the proposed adaptive model identification method is shown in Fig. 4. The proposed adaptive identification starts by collecting a few data points till time step $T$ as shown in step-A of Fig. 4. The acquired data set is divided into two parts. The first part indicated by the blue box has $N_{t}$ data points to be used for identification using semi-blind robust system identification. The second part shown with the pink box has $M_{t}$ data points equal to the order of the reduced order model, to be used by the model (In)validation framework discussed in the previous section. For example, if the required reduced model order is $3\text{rd}$order model then only three data points are required for model (In)validation. However, if the $3\text{rd}$ order model is not validated and a reduced model of order 5 or 6 is used then five or six data points are required for model (In)validation. At time step $T$, the patient model is identified and the model is tested for model (In)validation which completes step-A.

At the $T+1$ time step, a new data point (green) has been acquired shown in step B. In step-C, the model (In)validation window has moved forward to include the new data point in the window leaving a past data point (black) available to be used for identification in the future if needed. The model identified in step-A is tested on this new model (In)validation window. Now, there can be two outcomes: (i) the Model is validated, i.e model is suitable for future predictions and controller design or the model is invalid for future predictions and controller design. If the model is validated then step-E in Fig. 4 is followed. (ii) if the model is (In)validated then step-D is followed, where the model identification window has expanded to include the extra available data point (black) in the window which makes the total data points equal to $N_{t} + 1$ available for re-identification. The model is updated by re-identification through semi-blind robust system identification. The newly identified model is tested on the model (In)validation window before using it for prediction. This procedure is followed recursively at each time step.

The practical implementation of the aforementioned adaptive individualized patient modeling framework to develop personalized patient models which represent patients' true dose-response characteristics is given in Algorithm 1. The user input to initialize the algorithm is the patient data which includes patient ID and patient-specific dose-response data, stability margin $r$, the minimum number of patient's clinical data for the identification $N_{t}$, initial and maximum acceptable reduced model order, $O$ and $O_{max}$, respectively. The full-order model is identified in steps 1-2. Step 3 reduced the model order based on the initial value of $O$. The identified individualized patient model is processed through model (In)validation, as discussed in Section III, at step 6 after selecting the data points $M_{t}$ to be used for model (In)validation at step 5. The amount of data points $M_{t}$ is equal to the coefficients of the reduced-order model obtained in step 4.

If the model (In)validation requirements are met, the algorithm shows the verified personalized patient model. Otherwise, the model is not suitable for prediction and controller design, and the reduced model order, $O$, is raised for the same value of $r$. $r$ is raised if the order of the model is higher than $O_{max}$. The amount of data points, $N_{t}$, utilized for the identification process is increased if $r>r_{max}$, and the algorithm returns to step 1.

It is important to mention that for model adaptation through re-identification, all the past available data points are used, however, for first model identification only minimum $N_{t}$ data points are used. Although the maximum low-order model limit can be extended, low-order models are favored for controller design since they have fewer parameters to adjust. Low-order models have also been favored in clinical applications due to the ease with which the resulting models can be explained.

Algorithm 1: **Adaptive individualized patient dose-response modelling**
**Input:** Patient-ID, warfarin-INR clinical data, $N_{t}$, $r_{max}$, $O_{max}$.
**Output:** Discrete-time individualized patient model, $G_{r}$
1 **function** Semiblind($N_{t}$, $r$, warfarin-INR clinical data, ID);
2 **return**(G)
3 **function** ReduceModelorder(G, O);
4 **return**($G_{r}$)
5 Select, $M_{t}$, data points for model (In)validation.
6 **function** ModelInvalidation($G_{r},M_{t}$);
7 **if** $\Vert \Delta \Vert _\infty < 1$ **then**
8 **print**(model validated.)
9 **return**($G_{r}$)
10 **else**
11 **print**(model invalidated.)
12 **If** $O< O_{max}$ **then**
13 $O=O+1$; **repeat step 4.**
14 **else**
15 **if** $r< r_{max}$
16 Set default $O$; Increase $r$;
17 **repeat step 2.**
18 **else**
19 Set default $O$ and $r$; Increase $N_{t}$;
20 **repeat step 2.**
21 **endif**
22 **endif**
23 **endif**
24 **exit**

## V. Results

This section shows the one-step-ahead prediction results of personalized patient models produced with and without model (In)validation using the semi-blind robust model identification approach. The MMSE between predicted and clinically obtained INR levels is used to assess the model's accuracy and also used to compare the performance of patient models with and without a model (In)validation. The clinical data of warfarin-INR of forty-four patients were gathered at the Robley Rex Veterans Administration Medical Center. Dr. Brier (mentioned in the acknowledgment) was funded by the Department of Veterans Affairs to look at the pharmacogenetics of warfarin. This dataset consists of dose and INR data from patients collected from 2008-2012. Dose and INR data were abstracted from pharmacy records for all subjects, additionally, using an informed consent process, genetic information was determined for CYP2C9 and VKORC1. Data provided for this project will be an incrementing time field starting a 1 (day), dose schedule (ie 5 mg MWF, 7.5 mg TThSaSu), INR, and CYP2C9 and VKORC1 status. The Human Subjects Protection Program Office at the University of Louisville has given this project IRB approval. The data set contains patient ID, warfarin dosage, and INR values. Identifying patient models at the earliest stages of treatment is desired for precise drug delivery. It is worth mentioning that model identification with model (In)validation requires some extra data points depending on the order of the identified model.

In the following results for the model (In)validation process, the model order is selected which satisfies the conditions of model (In)validation, $\Vert \Delta \Vert _\infty < 1$, ranging between $3\text{rd}$ and $5\text{th}$ order models. However, for models identified without model (In)validation, we will be using the Akaike Information Criterion, $AIC=2\,K \times 2ln(L)$, to select the appropriate model order. Here, $L$ is the log-likelihood of the model best fit and $K$ is the independent variable of the model. As there is no adaptation involved in models identified without model (In)validation, the model order will not change once selected through the Akaike information criterion. For the comparison with the existing methods, we have used Kalman filter-based ARX and ARMAX recursive models from MATLAB system identification toolbox [42], [43], [44].

**Fig. 5.**
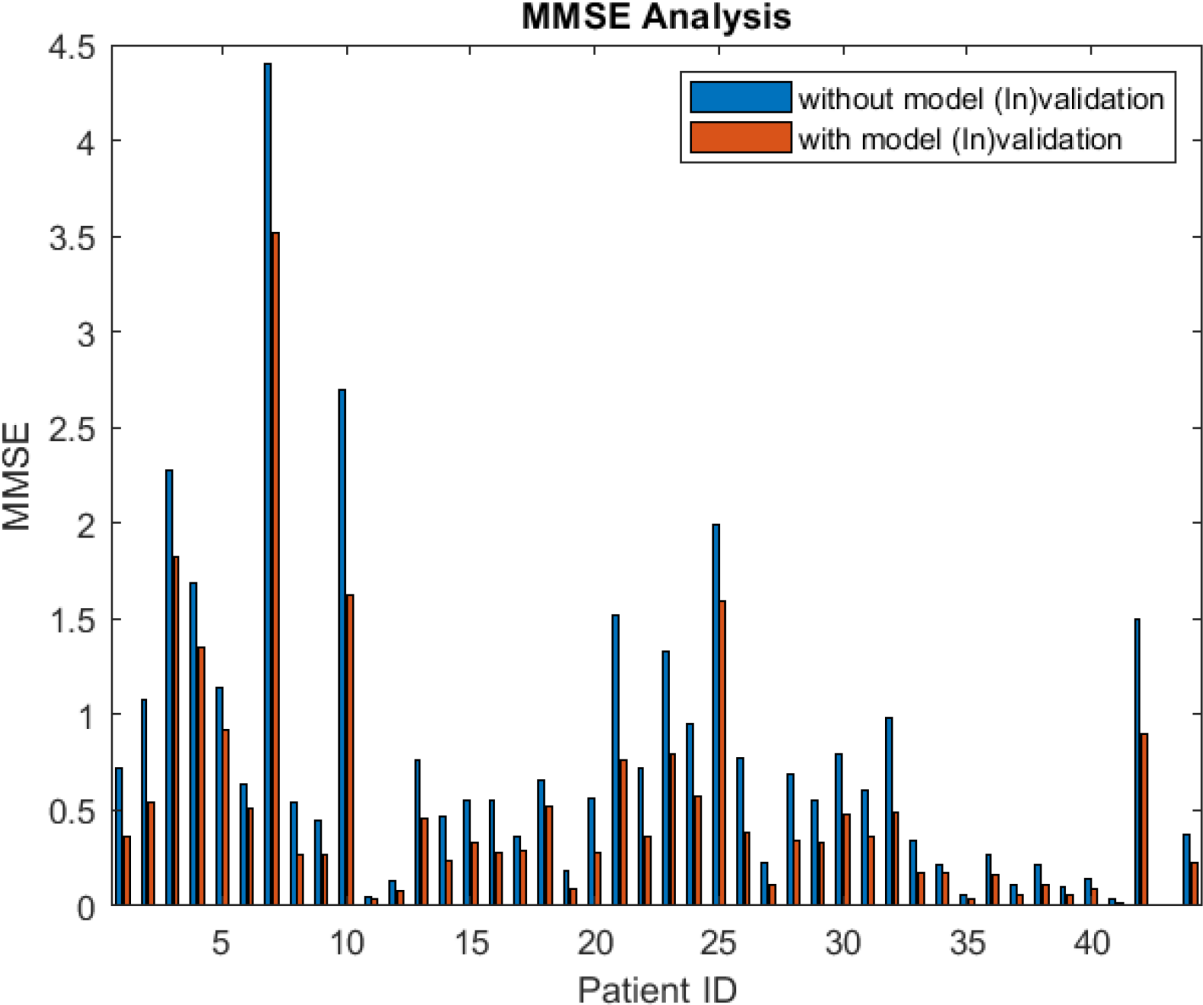
Error comparison of models obtained with and without model (In)validation.

Fig. 5 shows the MMSE results of the identified model for each patient. The blue bars show the MMSE values of models identified without model (In)validation and the black textured bars show the MMSE value of models identified with model (In)validation. The figure shows that MMSE values for models identified with model (In)validation are less than without model (In)validation. It shows that the prediction capabilities of the model increase along with confidence in the model to be used for controller design. To analyze these patients, we are showing the one-step-ahead prediction results obtained by the identified models of patients 7, 3, and 10. In these prediction results, we show the comparison between models identified with and without model (In)validation.

**Fig. 6.**
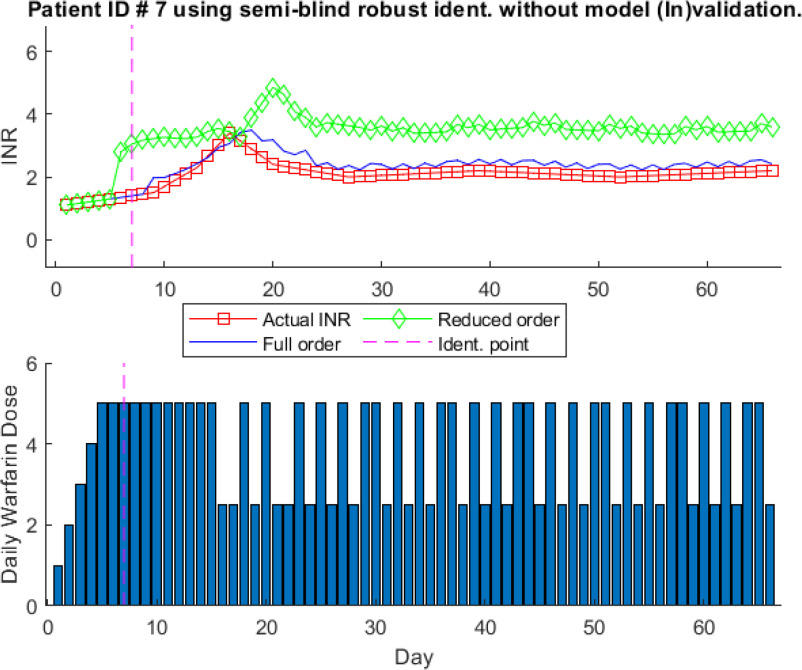
One-step-ahead prediction results using model identification without model (In)validation for patient ID # 7.

Fig. 6 shows the one-step-ahead prediction results for patient # 7 with $4\text{th}$ order model shown in (9) identified using semi-blind robust system identification without model (In)validation process.

\begin{equation*}
G_{7}(z)=\frac{ 0.09 z^{4} + 0.13 z^{3} + 0.12 z^{2} + 0.08 z + 0.001}{z^{4} + 0.50 z^{3} - 0.09 z^{2} - 0.45 z - 0.86} \tag{9}
\end{equation*}

**Fig. 7.**
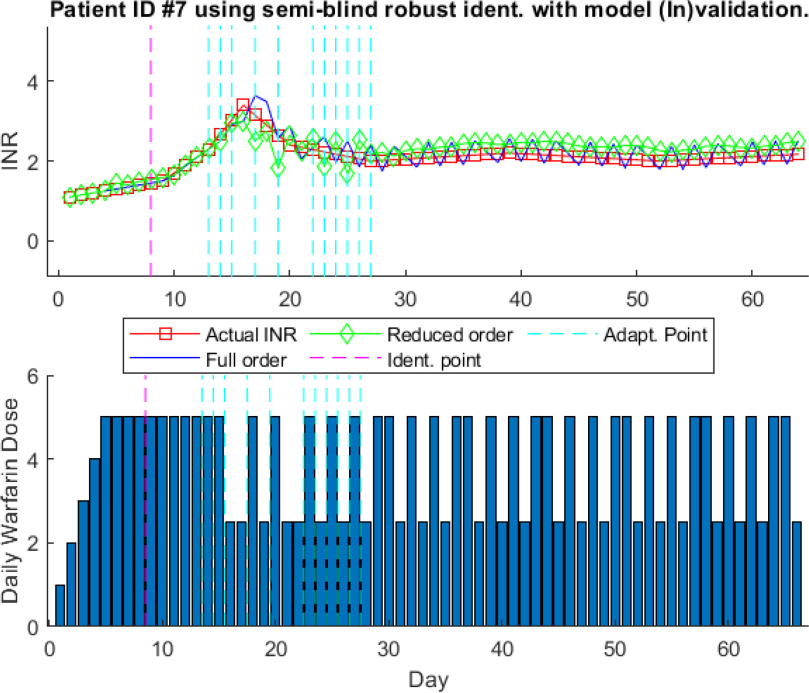
One-step-ahead prediction results using model identification with model (In)validation for patient ID # 7.

The clinically obtained actual INR levels and warfarin dosages are shown by a red line with squared markers and blue bars (bottom), respectively. The predicted INR levels, in response to clinically obtained warfarin dosage, using identified full-order model and reduced-order model are shown with a blue line and green line with diamond markers, respectively. The model is identified using seven data points, $N_{t} =7$, highlighted with a pink dotted vertical line. It can be seen that identified $4\text{th}$ order model is not able to completely mimic the clinically obtained INR values. However, the $6\text{th}$ order model, equal to one less of the data points used for identification, can predict the INR values close to actual INR values with slight error. It shows that this identified model is not able to predict the does-response with better accuracy. Therefore, we observe the prediction results of the patient # 7, however, this time we introduce the model (In)validation process along with semi-blind robust system identification. Similar to Fig. 6 seven data points are used for initial identification and the model is processed through model (In)validation as shown in Fig. 7 and mathematical model structure is shown in (10).

\begin{equation*}
\begin{split} G_{7}(z) =\left\lbrace \begin{matrix}\frac{0.12 z^{3} - 0.13 z^{2} + 0.04 z - 2.677e^{-5}}{z^{3} - 1.48 z^{2} + 0.5 z - 0.008}& 8 {\leq} n{\leq} 12\\
 \frac{0.064 z^{4} - 0.122 z^{3} + 0.09 z^{2} - 0.023 z + 4.012e^{-3}}{z^{4} - 2.75 z^{3} + 2.81 z^{2} - 1.18 z + 0.13} & n= 13 \\
 \vdots & \vdots \\
 \frac{0.03 z^{5} + 0.05 z^{4} + 0.04 z^{3} + 0.01 z^{2} - 0.02 z - 0.013}{z^{5} + 0.42 z^{4} - 0.45 z^{3} - 0.82 z^{2} - 0.49 z + 0.4}& 27 {\leq} n{\leq} 64 \end{matrix}\right. \end{split} \tag{10}
\end{equation*}

Fig. 7 shows that the model is adapted multiples times (eleven times), represented with the vertical dotted cyan line. This shows that the previously identified model is not valid and it is needed to be updated through re-identification using semi-blind robust system identification method. For patient # 7, there will be twelve model equations as the model is updated eleven times within sixty-four days of treatment. It can be seen that model identified with model (In)validation predicts the INR values close to clinically obtained INR values. This highlights the benefit of the recursive adaptive model identification algorithm to increase the accuracy of the model prediction. It is interesting to note that after the thirty data points, there is no need for model adaptation. This illustrates that with the passage of time and the arrival of new data, the adaptive algorithm has identified a model which can define the dose response of the patient at later stages as all the variations to be experienced in the latter part of the treatment are already captured by the model.

For the comparison, recursive ARX and ARMAX models with Kalman filter are used. 6$\text{th}$ model order is used for ARX and ARMAX model for fair comparison since the full order model for model (In)validation-based adaptive model identification is 6$\text{th}$ order using 7 data points for initial identification and reduced model varies throughout the treatment. It is important to mention that model structure and model order are predefined for ARX and ARMAX model identification. Kalman filter-based recursive system identification updates the model coefficients based on the error between predicted output and actual output.

**Fig. 8.**
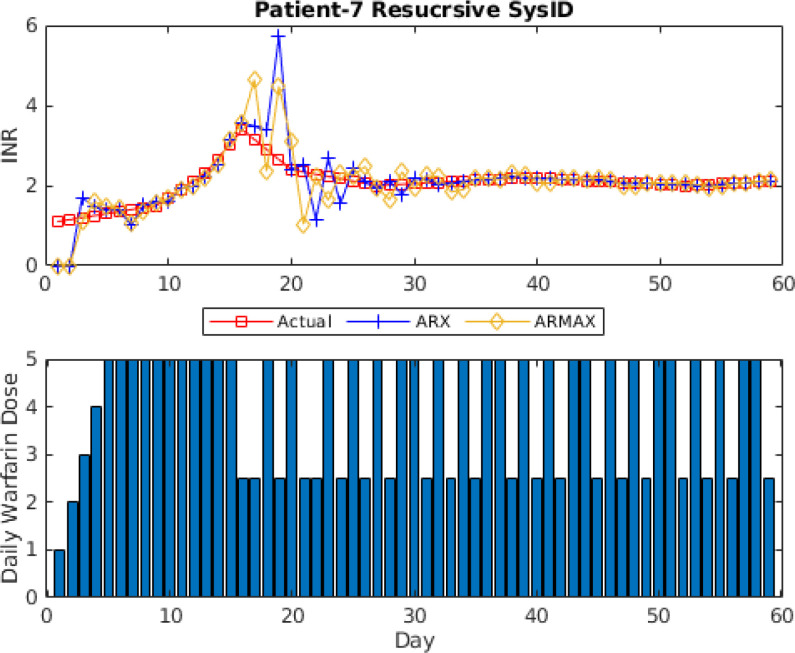
One-step-ahead prediction results using recursive ARX and ARMAX models for patient ID # 7.

Fig. 8 shows the prediction results for ARX and ARMAX based recursive system identification patient ID #7. The identified models can reduce the prediction error with 3 data points. However, the recursive algorithm suffered performance degradation from 15 to 25 days of treatment. It is mainly due to a change in warfarin dosage which increased the prediction error. However, the recursive identification algorithm can reduce the prediction error within 10 time steps and find a stable model to avoid this kind of performance degradation in the future. It can be seen by the comparison of Figs. 7 and 8 that ARX and ARMAX have the advantage of quickly identifying suitable models given that appropriate model structure and model order are predefined. On the other hand, semi-blind robust model identification with model (In)validation can find a patient model using 7 data points and adapt the model as treatment progresses with low prediction while handling the changes in dosing habits.

**Fig. 9.**
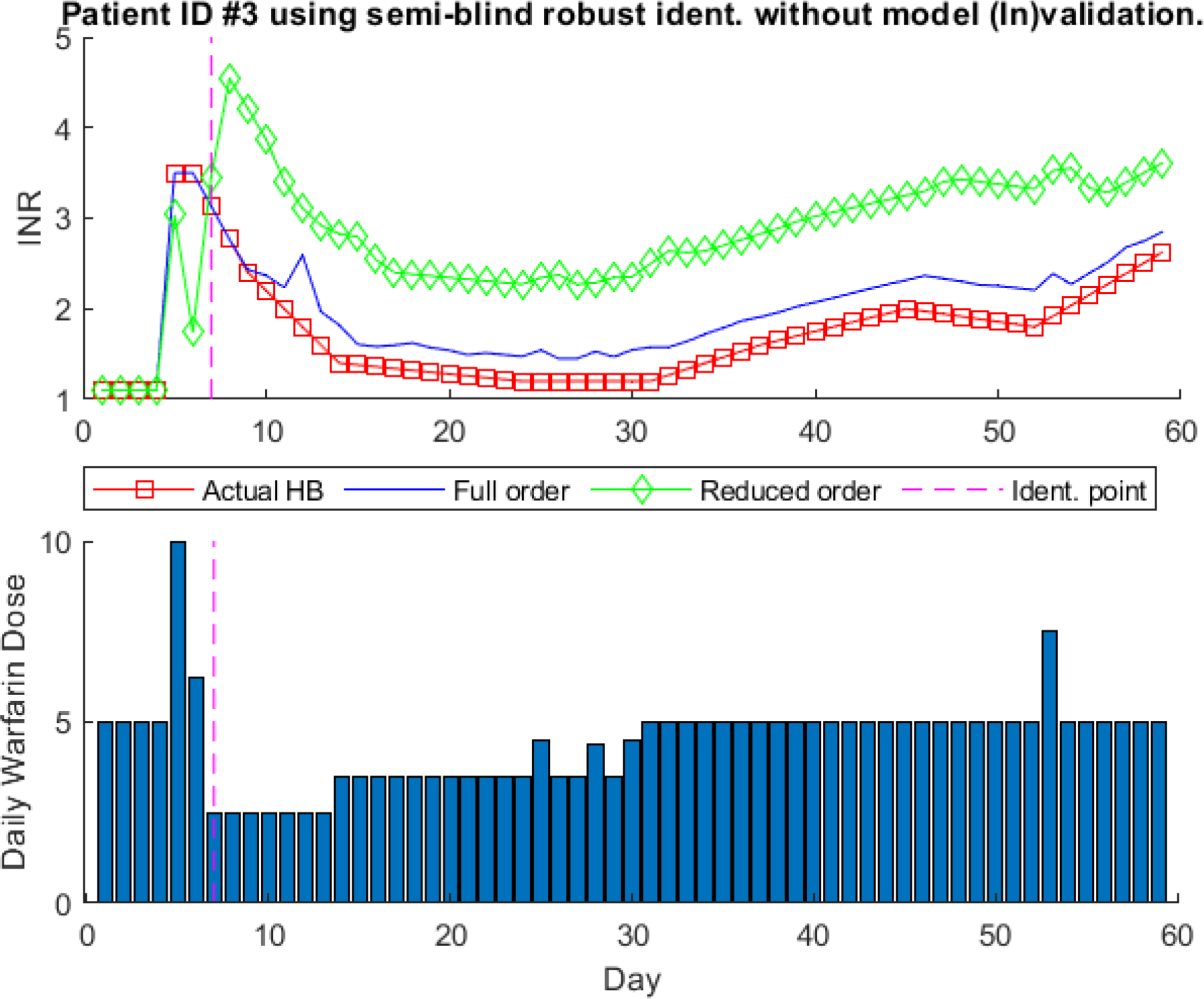
One-step-ahead prediction results using Semi-blind without model invalidation for patient ID # 3.

Furthermore, one-step-ahead prediction results for patient # 3 based on model identification without model (In)validation are shown in Fig. 9. The mathematical model identified without model (In)validation is shown in (11).

\begin{equation*}
G_{3}(z)\!=\!\frac{ 0.09 z^{5} \!+\! 0.02 z^{4} \!+\! 0.003 z^{3} \!+\! 0.02 z^{2} \!+\! 0.05 z \!-\! 2e^{-4} }{z^{5} - 0.78 z^{4} - 0.17 z^{3} + 0.16 z^{2} + 0.31 z - 0.49} \tag{11}
\end{equation*}

For model identification without model (In)validation, 5$\text{th}$ order model is used based on $AIC$. It is shown by Fig. 9 that the reduced order model has a higher prediction error as compared to the full order model, which is $6\text{th}$ order model equal to one less of data points used for model identification. To improve the prediction results, (12) shows the mathematical model for patient #3 obtained using model (In)validation-based proposed adaptive identification algorithm.

\begin{equation*}
 G_{3}(z){=}\left\lbrace \begin{matrix}\!\frac{0.1356 z^{3} - 0.1867 z^{2} + 0.1011 z - 0.01912}{ z^{3} - 1.827 z^{2} + 1.024 z - 0.1909}& 8 \!\leq\! n\!\leq\! 12\\
 \!\frac{0.0697 z^{3} + 0.01878 z^{2} - 0.002374 z - 0.04851}{z^{3} - 1.044 z^{2} + 0.1159 z - 0.05208} & 13 \!\leq\! n\!\leq\! 14 \\
\! \vdots & \vdots \\
\! \frac{0.02 z^{5} + 0.069 z^{4} + 0.059 z^{3} - 0.02 z^{2} - 0.04 z - 0.01}{z^{5} + 1.54 z^{4} - 0.52 z^{3} - 1.6 z^{2} - 0.43 z + 0.07}& 53\!\leq\! n\!\leq\! 59 \end{matrix}\right. \tag{12}
\end{equation*}

**Fig. 10.**
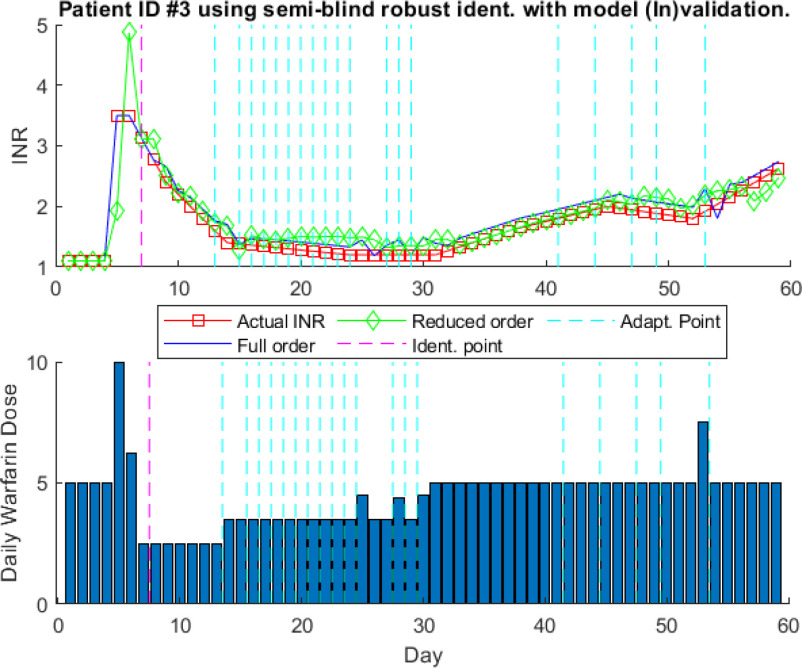
One-step-ahead prediction results using Semi-blind with model invalidation for patient ID # 3.

**Fig. 11.**
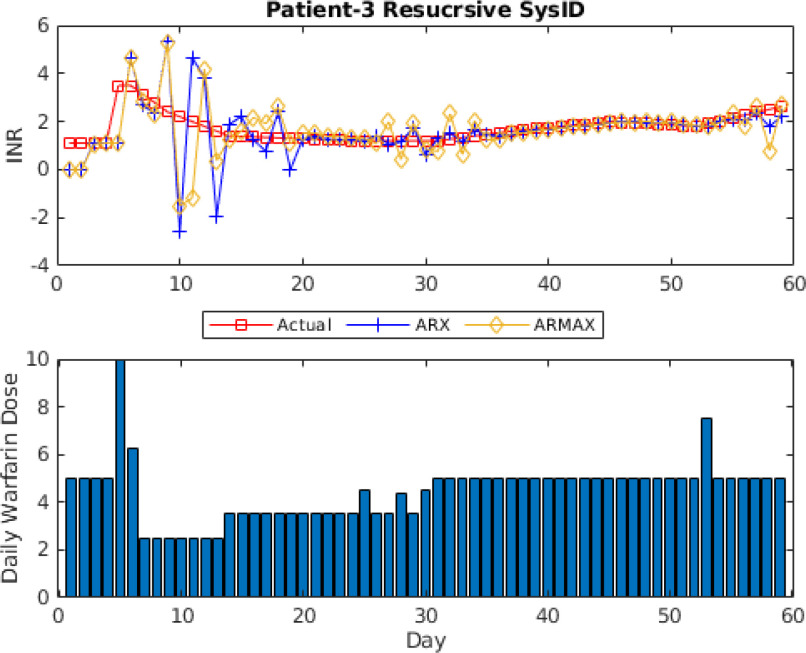
One-step-ahead prediction results using recursive ARX and ARMAX models for patient ID # 3.

Fig. 10 shows prediction results for this model. The initial model is identified using 7 data points. The initial reduced model, selected by the adaptive algorithm discussed in Section IV, is 3$\text{rd}$ order model. It can be seen that model is updated multiple times to reduce the prediction error throughout the treatment. It shows the effect of the model (In)validation-based model adaptation. Fig. 11 shows the prediction results of recursive ARX and ARMAX model identification for patient ID #3. It can be seen by comparison of Figs. 10 and 11 that the proposed adaptive algorithm can provide the patient model with low prediction error without performance degradation due to changes in dosages. Recursive ARX and ARMAX models show good prediction results. However, these models have higher prediction errors due to changes in dosages which is avoided in the proposed adaptive algorithm.

**Fig. 12.**
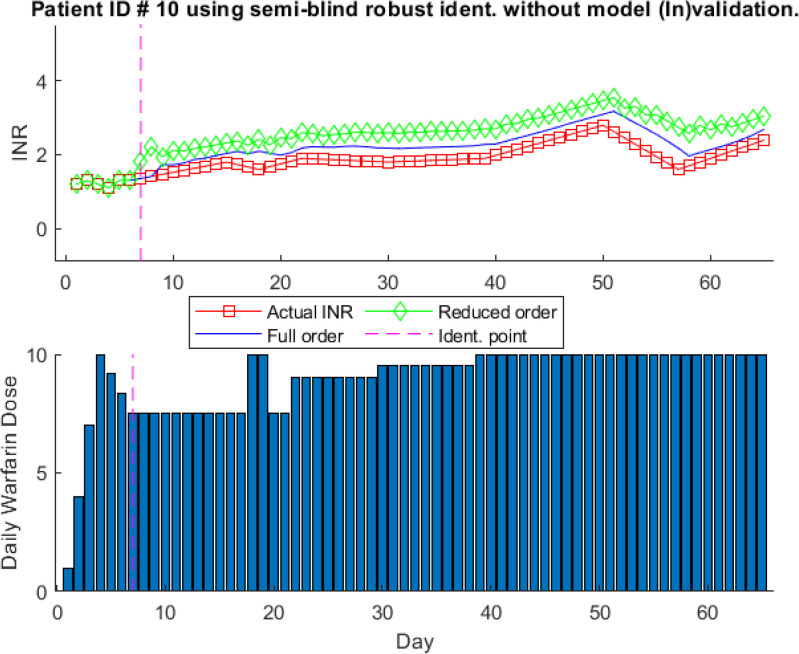
One-step-ahead prediction results using model identification without model (In)validation for patient ID # 10.

Fig. 12 shows a one-step-ahead prediction for the model identified without model (In)validation for patient ID # 10. Here, seven data points and $5\text{th}$ order model, selected based on Akaike Information Criterion, is used for semi-blind robust model identification as shown in (13). It can be seen that identified model predicted the INR values has some error. It can be seen that predicted INR values using the full order model, shown in the solid blue line, are off as well.

\begin{equation*}
G_{10}(z)\!=\!\frac{ 0.1 z^{5} \!-\! 0.02 z^{4} \!+\! 0.04 z^{3} \!-\! 0.01 z^{2} \!+\! 0.03 z \!+\! 0.8e^{-3}}{z^{5} - 1.4 z^{4} + 1.28 z^{3} - 1.09 z^{2} + 0.9 z - 0.62} \tag{13}
\end{equation*}

**Fig. 13.**
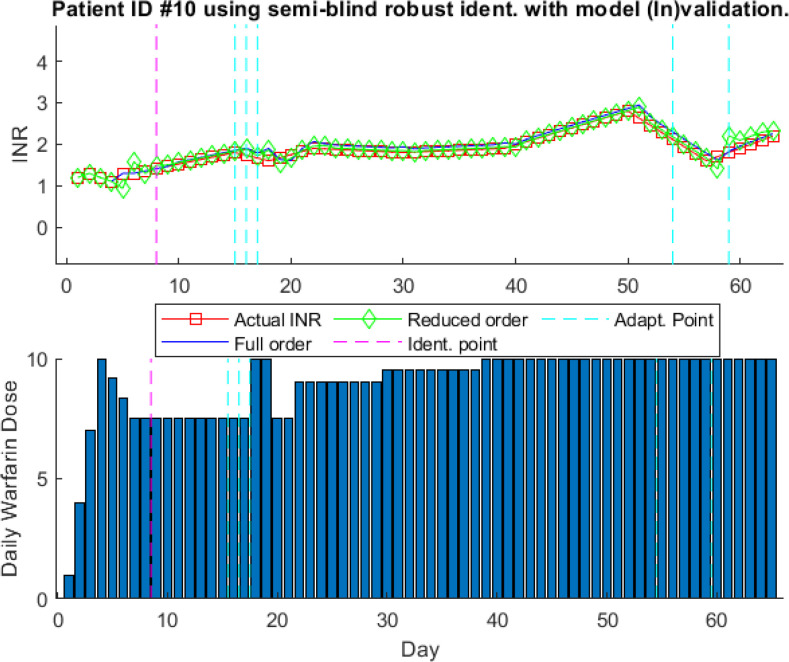
One-step-ahead prediction results using model identification with model (In)validation for patient ID # 10.

For comparison, we include the model (In)validation in the identification process to use the adaptive model identification algorithm for patient # 10 as shown in (14). The prediction results with model (In)validation for patient # 10 are shown in Fig. 13. The model is updated four times within sixty days of treatment, which shows that finding the model for patient # 10 is easier than for patient # 3. It can be seen that model adaptation increases prediction accuracy by capturing all the fluctuations. It is interesting to note that model is updated at the last period of treatment. During the last period of treatment clinically obtained warfarin dosages are constant. However, INR values are changing which makes the model to be updated. This highlights the need for model (In)validation to be introduced in the model identification process to increase the prediction accuracy and reliability of the model for controller design in the event of a change in dose-response characteristics.

\begin{align*}
 &G_{10}(z)\\
&=\left\lbrace \begin{matrix}\frac{0.07 z^{3} \!-\! 0.1 z^{2} \!+\! 0.06 z \!-\! 0.009826}{z^{3} \!-\! 1.9 z^{2} \!+\! 1.1 z \!-\! 0.21}& 8 \!\leq\! n\!\leq\! 14\\
 \frac{0.04 z^{3} \!-\! 0.05 z^{2} \!+\! 0.02 z \!-\! 0.001499}{z^{3} \!-\! 1.9 z^{2} \!+\! z \!-\! 0.1} & n= 15 \\
 \vdots & \vdots \\
 \frac{0.01 z^{5} + 0.01 z^{4} + 0.001 z^{3} - 1.7e^{-05} z^{2} - 0.01 z - 0.002}{ z^{5} - 0.4 z^{4} - 0.4 z^{3} - 0.3 z^{2} - 0.63 z + 0.7}& 59 \!\leq\! n\!\leq\! 63 \end{matrix}\right. \tag{14}
\end{align*}

**Fig. 14.**
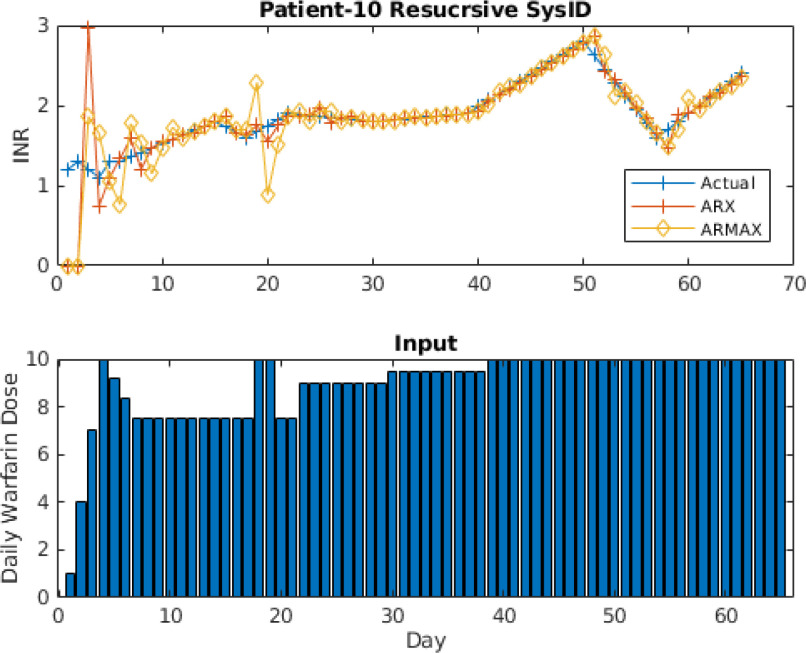
One-step-ahead prediction results using recursive ARX and ARMAX models for patient ID # 10.

Fig. 14 shows the prediction results for patient #10 for 6$\text{th}$ order recursive ARX and ARMAX models using the Kalman filter. The model order is chosen based on AIC and fair comparison with full-order models used by the proposed adaptive model identification algorithm. Fig. 14 shows that ARX and ARMAX models predicted the INR values very close to actual INR values, however, both models suffered higher prediction error around 5$\text{th}$ week and around 20$\text{th}$ week due to change in warfarin dosage. However, both recursive models can reduce the prediction error quickly.

The average error for all patients for the proposed method and recursive ARX and ARMAX models is shown in Table I. It shows that the overall prediction error for the proposed method is low than ARX and ARMAX models in the application of warfarin management. The results show that the Kalman filter based recursive model identification method has the advantage of quickly finding the patient model and handling the effects of changing characteristics. However, it suffers performance degradation due to changes in patient dose response and requires predefined model order and model structure. On the other hand, the semi-blind model identification method with model (In)validation can handle dose-response variations and update the patient model with low prediction error.

**TABLE I:** MMSE Comparison Between Identification Methods for Warfarin Management

Identification Method	MMSE value
Semi-blind without model (In)validation	2.1 $\pm$ 0.5
Semi-blind with model (In)validation	0.4 $\pm$ 0.3
Recursive ARX with Kalman Filter	0.5 $\pm$ 0.25
Recursive ARMAX with Kalman Filter	0.9 $\pm$ 0.4

## VI. Conclusion

Warfarin is an anticoagulant that is prescribed to patients suffering from thrombotic events and blood clotting. The effect of warfarin is measured by INR level. The desired range of INR levels is 2-3. The inappropriate dosage of warfarin can lead to the risk of bleeding and other health issues. It is therefore advantageous to administer warfarin using a robust and patient-specific method to address the inter-and intra-patient variability among patients. This paper aims to assist clinicians to predict the appropriate warfarin dosage by providing a robust and adaptive modeling framework for the individualized patient models using a limited number of clinical patient data.

To address these needs, we present a semi-blind robust model identification technique for warfarin dosage prediction. The semi-blind robust model identification takes the effect of medical history by using non-zero initial conditions to reduce the identification error. Furthermore, the model (In)validation framework is discussed to consistently monitor the identified patient model for model adaptation.

Through rigorous simulations, it is demonstrated that the proposed model identification and adaptation technique can provide models with high accuracy and alert the clinician in time if the model is no longer valid for prediction and controller design. For simulations, the clinical data of forty-four patients has been collected from the Robley Rex Veterans Administration Medical Center, Louisville. By calculating MMSE values between predicted INR level and clinically acquired INR data, it is shown that models obtained with the model (In)validation can help in dosage prediction with better accuracy as compared to the models obtained without the model (In)validation. The proposed adaptive model identification algorithm is also compared with recursive ARX and ARMAX model identification for warfarin management. The results show that recursive ARX and ARMAX models can predict INR values with low prediction errors. However, these models suffer higher prediction error when dose-response characteristics changes, while the proposed method can handle these changes with low prediction error. The goal for the future is to develop a controller based on the personalized models recognized by the semi-blind robust identification approach.
